# A study on innovation resistance of artificial intelligence voice assistants based on privacy infringement and risk perception

**DOI:** 10.1371/journal.pone.0320431

**Published:** 2025-04-08

**Authors:** Shanshan Liu, Yongseok Cheon, Jong-Yoon Lee, Minglu Wang, Ning Liu

**Affiliations:** 1 School of Communication, Nanyang Institute of Technology, Nanyang, Henan, China; 2 Research and Investifation Department, Korea Out of Home Advertising Center, Seoul, Republic of Korea,; 3 School of Art, Sangmyung University, Cheonan, Republic of Korea; 4 School of Art, Qilu University of Technology, Jinan, Shandong, China; 5 School of Journalism and Communication, Shanghai University, Shanghai, China; King Khalid University, SAUDI ARABIA

## Abstract

As a vital tool for human-computer interaction, artificial intelligence (AI) voice assistants have become an integral part of individuals’ everyday routines. However, there are still a series of problems caused by privacy violations in current use. This research aims to explore users’ risk perceptions and innovation resistance arising from privacy concerns when utilizing AI voice assistants. Descriptive statistics and correlation analysis were conducted using SPSS21.0 software to examine each variable. The mediating and moderating effects were analyzed through specific models provided by PROCESS. The findings of this research indicate that perceptions of risk serve as a mediator in the relationship between privacy violations and resistance to innovation. This elucidates the indirect pathway through which privacy concerns impact opposition to new technologies. Furthermore, the study reveals that anthropomorphism and informativeness can mitigate the perceived risks associated with AI voice assistants, consequently reducing resistance to innovation. By focusing on user psychology, this study offers valuable insights for the development and enhancement of AI voice assistants, underscoring the importance of addressing user concerns regarding privacy and risk.

## 1. Introduction

Due to advancements in modern technology, human existence has progressively shifted into the digital space, with a plethora of sophisticated applications being created through the blend of state-of-the-art intelligent technologies, including artificial intelligence (AI), big data analysis, machine learning, business intelligence, and the Internet. Among these innovations, artificial intelligence serves as a pivotal force in automating various sectors, providing the ability to learn and make optimal real-time decisions. The swift evolution of artificial intelligence and machine learning (ML) has enabled machines to learn independently. AI has revolutionized multiple industries, such as finance, healthcare, retail, manufacturing, and media, and continues to explore new areas [1].

Intelligent voice assistants have become an essential part of people’s daily lives, serving as a means of interacting with computers. They offer various functions, such as speech recognition, semantic understanding, dialogue management, and speech synthesis. These extensive capabilities greatly enhance the convenience and enjoyment of our lives. Moreover, in addition to the fundamental features of speech recognition and semantic interpretation, they also encompass basic schedule management and reminders, voice translation, personalized customization, music playback, and movie queries [2]. Smart speakers, in particular, not only serve as virtual personal secretaries but also function as speakers for radio and music playback, providing consumers with a more practical and entertaining experience. Furthermore, they include features such as weather and travel updates, notifications, chat functions, and control of IoT devices [3], thereby revolutionizing the way we interact with technology. As a result, AI voice assistants have become an indispensable tool in our daily lives. It can be seen that the rapid advancement of information technology has revolutionized various aspects of our lives, making it easier than ever to collect and store vast amounts of data. One significant change resulting from this development is the rise of user information databases. These databases store personal information, preferences, and behaviors of individuals, which have become invaluable resources for businesses aiming to enhance their marketing strategies. Consequently, user information is now considered one of the most crucial assets for any company seeking to stay ahead of the competition. However, the collection and analysis of such large volumes of user data also raise important concerns about privacy infringement. With the increasing sophistication of artificial intelligence and machine learning, machines can now provide personalized recommendations and communicate through social robots. This has further intensified the demand for more user data, leading to an increasing number of cases where individuals’ privacy has been violated. The issue of online privacy infringement has gained significant attention in recent years, particularly due to the rise of social media platforms and the increasing number of data breaches. According to a survey conducted by the Public Opinion Laboratory of the Chinese Academy of Social Sciences, the protection of personal privacy is considered the primary concern in China’s Internet governance [4]. This is a consequence of the expanding freedom of data flow, which has resulted in a higher risk of personal privacy disclosure [5]. As more and more cases of social privacy infringement come to light, users have become increasingly aware of the importance of protecting their personal information. This has led to a growing resistance against various forms of privacy infringement, which now acts as a limiting factor for the development of smart media. Companies must now find ways to strike a balance between the need for user data and the desire for privacy protection.

This study aims to investigate the risk perceptions and innovation resistance caused by users’ concerns about privacy invasion when using AI voice assistants. Because AI voice assistants may expose sensitive personal information when used. In this study, risk perception serves as a mediating variable, while personification and informationality are considered moderating variables. The objective is to test whether the corresponding mediating effects, moderating effects, and moderated mediating effects are established. We anticipate more comprehensive technical support in the future to minimize or eliminate the perception of risk when using AI voice assistants. Hope that this research will serve as a valuable reference for the design and development of AI voice assistants.

## 2. Theoretical background

### 2.1. Innovation resistance

Innovation resistance, as defined by Ram (1987), pertains to the resistance encountered by consumers when faced with change caused by innovation. This resistance is a natural response since innovation inherently brings about change [6]. Conversely, anti-innovation does not signify a negative stance towards innovation itself, but rather, it represents resistance to the changes brought about by innovation. Therefore, anti-innovation does not oppose the notion of accepting innovation but rather signifies a variation in its degree. It is a natural mental state experienced by consumers during the process of acceptance [6]. This resistance arises due to the potential impediment of existing circumstances by the changes accompanying innovation. The current bias theory, presented by Samuelson & Zeck, posits that consumers tend to resist novel changes and instead prefer the status quo due to their inherent aversion to change [7]. Within the domain of consumer behavior, theories such as the phenomenal deviation theory and cognitive risk theory have been proposed to elucidate innovative resistance. The current bias theory, proposed by Samuelson & Zech, supports the idea that consumers resist new changes and favor traditional methods due to their inherent bias against change. Several theories propose that factors influencing this bias are associated with risk aversion, perceptual biases, and emotional attachments [7]. The risk perception theory examines how individuals behave in circumstances where the outcome of a decision is uncertain. By considering the utility and perceived level of risk, rational consumers assess whether to adopt innovation [7]. When the utility outweighs the perceived risk, consumers are more inclined to embrace innovation [8]. Ultimately, these psychological factors play a significant role in fostering resistance to innovation during the acceptance process.

In the study by Sheth (1981), the importance of considering an individual’s psychological state when examining resistance to innovation is emphasized. Sheth introduces two psychological concepts that provide insight into why individuals resist innovation. The first concept, called ‘habit of existing biases,’ refers to situations where innovation conflicts with established ways of life, leading to reluctance and resistance. The second concept, known as ‘perceived risk,’ encompasses concerns about potential negative consequences, uncertainty about the innovation’s performance, and anticipated side effects [9]. To successfully develop and implement innovations, it is suggested that knowledge about consumer resistance be gathered and utilized in advance. This involves understanding the psychology of individuals and their potential resistance to innovation rather than exposing them to innovation without prior comprehension. When consumers perceive something as an innovation, it can trigger resistance due to its new and unfamiliar nature, which can vary based on their psychological characteristics [6]. According to Talke and Heidenreich (2014), an individual’s resistance to change is influenced by various personality traits, including sensation-seeking and openness to experience [10]. Furthermore, Rogers (2003) emphasizes that a positive attitude toward change and self-efficacy are crucial characteristics for an individual’s acceptance of innovation [11]. When individuals perceive high levels of risk associated with innovation, they are more likely to resist it. Therefore, it is crucial to understand and address these psychological concepts in order to manage resistance to innovation.

### 2.2. Privacy infringement and risk perception

The definition of privacy varies across different fields. Initially, privacy was described as ‘the right to be alone, free from external intrusion and interference’ [12]. However, with the advancement of information and communication technology, the concept of privacy has evolved. Issues such as personal information leakage and invasion of privacy have become increasingly serious, leading to heightened consumer concerns. As a result, research on privacy issues has deepened, and the definition of privacy has expanded to encompass various domains, including management, economics, psychology, sociology, and others [13–18]. Furthermore, Dinev et al (2006) defined privacy leakage worry as ‘the spontaneous and involuntary exposure of personal information online’ and characterized it as ‘the concern regarding the loss of users’ personal information’ [19]. Featherman et al (2003) defined privacy danger as the inadvertent exposure of personal information and the potential loss of control due to the extent of disclosure [20]. This means that when individuals access online information, there have been instances of privacy violations where personal information is collected and used without their consent. As a result, the original definition of privacy has evolved to include the concept of information privacy [21]. According to the literature, online privacy can be categorized into three types: performance privacy, proximity privacy, and information privacy. Information privacy refers to the level of control individuals have over their personal information online [22]. It encompasses the right to make decisions about personal information, granting individuals an active role in controlling their own information.

Privacy concerns have been raised regarding personalization services that employ artificial intelligence. Even the inadvertent collection and aggregation of data can be seen as an intrusion into users’ privacy. Additionally, location-based services may be offered to analyze the user’s environment. A survey on privacy concerns related to location-based services (LBS) revealed that 24% of potential users express significant apprehension regarding the disclosure of their location information [23,24].

Utilizing personal information to enhance the functionality of intelligent personal secretaries offers convenience to users. However, it also raises concerns about potential privacy breaches if not handled with caution. Numerous previous studies have examined the issue of privacy in relation to smart personal secretaries. In his research on identifying factors that influence users’ acceptance of these assistants, Jongmin Lee (2019) identified privacy infringement as a significant factor that hampers usage intentions [25]. Subsequent discussions have further emphasized the importance of addressing privacy concerns. According to Kim Jae Hui (2010), the more information companies collect from users and the longer they accumulate it, the greater the risk of infringing on users’ private activities [26]. Hwansoo Li et al (2013) state that big data technology enables access, collection, and storage of personal information and can even analyze sensitive information [27]. This can lead to increased privacy concerns and resistance among people.

### 2.3. Anthropomorphism and informational of AI voice assistants

This study aims to examine the user experience of AI voice assistants and understand their cognitive structures regarding privacy violations. Additionally, it explores the potential impact of innovation resistance on future use and service recommendations. Therefore, it is crucial to consider the psychological changes that users may undergo.

Anthropomorphism can be defined as the attribution of human-like characteristics or mental states to actual and imagined non-human agents and things. In their 2007 paper, Epley et al. aimed to explain this phenomenon from a psychological perspective. They discussed the term anthropomorphism as explored in psychology and its definition [28]. Anthropomorphism refers to a concept that has been studied many times in fields such as marketing, communications, HCI, and robotics [29].

Anthropomorphism involves the assignment of human-like qualities and perspectives to objects that are not human. This concept is particularly relevant in the context of robots and interactive services. Previous studies have explored how users respond to products and services that are amenable to anthropomorphism. The findings reveal that greater degrees of anthropomorphism have a positive impact on user satisfaction and their inclination to persist in using an AI speaker through voice interaction [30].

There are three ways in which artificial objects can be subject to anthropomorphism: external anthropomorphism, internal anthropomorphism, and social anthropomorphism [31]. Anthropomorphism relevant to this article includes internal and social anthropomorphism. Internal anthropomorphism entails emphasizing the emotional and psychological dimensions of humans. Social anthropomorphism, on the other hand, pertains to the interaction between users and machines [32].

The investigation of the impact of anthropomorphism is gaining momentum in scientific research. However, the analysis of user characteristics and their usage patterns in relation to anthropomorphism remains insufficient. This research project seeks to explore the potential adaptation of the association between privacy violation and perception of risk in accordance with different degrees of anthropomorphism observed among users of smart voice assistants. Furthermore, it aims to assess whether modifying the level of anthropomorphism can diminish user perception of risk in situations involving privacy infringement.

The concept of information systems in marketing refers to the provision of relevant information that directly influences consumers’ purchase intention towards a product [33]. In the online environment, information is comprised of specific forms such as text, voice, video, and images, which consumers can access through various channels [34]. The intelligent recommendation function of big data and intelligent algorithms enables artificial intelligence voice assistants to offer consumers a personalized thinking space and a wealth of knowledge across various fields. However, some consumers may become restricted to obtaining information only within a specific field. This phenomenon, known as the ‘information cocoon room; can potentially create challenges for consumers [35]. Therefore, in addition to anthropomorphism, this study will explore the potential role of information in mediating and mitigating resistance to innovation in the presence of risk perceptions. Based on preliminary discussions, the following hypotheses and research questions were formulated:

Research Question 1: Does the level of perceived anthropomorphism while utilizing AI voice assistants moderate the relationship between privacy infringement and risk perception?

Research Question 2: Does the level of perceived informativeness while utilizing AI voice assistants moderate the relationship between risk perception and innovation resistance?

Hypothesis 1: The perception of risk during the process of using AI voice assistants will mediate the relationship between privacy infringement and innovation resistance.

**Fig 1:**
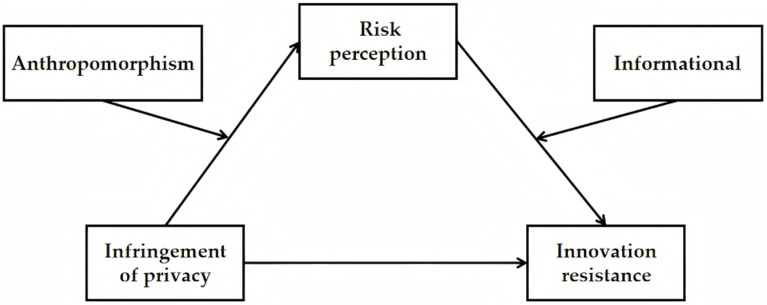
Research model.

To support the conceptualized structural model presented in this paper (Fig 1), we aim to investigate the following research questions and hypotheses.

## 3. Materials and methods

### 3.1. Participants

The study was conducted online using the Questionnaire Star platform, and the recruitment period for the survey began in December 2023 and ended in February 2024. The participants consisted of individuals within a wide age range, primarily between 18 and 39 years old, who possessed some knowledge and basic understanding of AI voice assistants, as well as a considerable level of internet access. These criteria fulfilled the research requirements of this investigation. Additionally, according to a survey conducted by PricewaterhouseCoopers (PWC), voice-assisted technology has a high adoption rate among individuals aged 18 to 24. However, the age demographic that utilizes AI voice assistants more frequently falls within the range of 25 to 49 years old, with 65% of this group being classified as ‘heavy’ users who issue voice commands to their devices at least once a day. A total of 436 completed questionnaires were received. In cases where users completed the questionnaires hastily or exhibited no relevant experience, their responses were deemed invalid and, therefore, excluded from the analysis. Consequently, a final count of 419 valid questionnaires was included in this study, with 213 from female participants and 206 from male participants. The variables employed in this investigation were operationally defined and measured using a seven-point Likert scale for each item.

### 3.2. Variable measurement

#### 3.2.1. Infringement of privacy.

Information privacy refers to the right to have exclusive control over personal information, including the right to participate in the flow and use of personal information [36]. It can be seen as a state that does not compromise one’s private life and does not disclose personal information at will. In this study, measured If AI voice assistants are used, it is worrying to know if anyone will collect information about themselves or their families; I am worried that using AI voice assistants may expose my personal life; People who value their private lives should refrain from using AI voice assistants; In order to use AI voice assistants smoothly, I believe that one should give up their personal life to a certain extent; In order to better use AI voice assistants, I believe a certain level of information should be provided about me; If privacy is excessively emphasized, it is impossible to use AI voice assistants, etc [37–39].

#### 3.2.2. Risk perception.

Risk perception is defined as the level of understanding of overall risk when using intelligent voice assistants, and modified and used the previously studied questions based on this study. I believe intelligent voice assistants have potential dangers; I believe intelligent voice assistants cannot prevent sensitive personal information from being leaked, and using intelligent voice assistants may violate privacy. Compared to other media platforms, intelligent voice assistants have more uncertainty, measured using a 7-point scale. The response options for the questionnaire ranged from 1 (strongly agree) to 7 (strongly disagree).

#### 3.2.3. Anthropomorphism.

Anthropomorphism involves the assignment of human-like qualities and perspectives to objects that are not human. This concept is particularly relevant in the context of robots and interactive services. In order to measure perceived anthropomorphism, the scale of indirect anthropomorphism measurement used in existing studies was used [40]. Kim & Sundar (2012) developed a scale that indirectly measures perceived anthropomorphism (Social, Personal, Friendly, Likable), taking into account the cognitive process of people’s unconscious anthropomorphism [40].

#### 3.2.4. Informational.

The scale revised by Yang was used in this study (e.g., intelligent voice assistants provide the information I need; intelligent voice assistants provide the information I want; intelligent voice assistants provide me with appropriate information. These questions were modified and measured using a Likert scale ranging from 1 (completely disagree) to 7 (strongly agree) [41]. The higher the score, the stronger the user’s affective responses.

#### 3.2.5. Innovation resistance.

All advancements give rise to opposition to modification as they necessitate individuals to accommodate [5]. In accordance with the research conducted by Zaltman & Wallendorf (1983), resistance to innovation pertains to any conduct that strives to uphold the existing state of affairs despite the pressure to change it [42]. The level of resistance to innovation can be influenced by user attributes such as perceptions, motivations, personality, previous encounters, and demographic factors. Although there might be variations in the characterization of innovation resistance within research, the non-adoption of novel products is universally acknowledged as a noteworthy attribute [43]. In this investigation, measurements were taken on indicators like ‘I believe AI voice assistants induce anxiety in individuals,’ ‘I would refrain from utilizing AI voice assistants,’ and ‘I am hesitant about using AI voice assistants.’

### 3.3. Procedure

This study was approved and reviewed by the Academic Research Ethics Committee of the Qilu University of Technology. Consent was obtained from the participants in accordance with academic and ethical requirements.

### 3.4. Data analysis

We used SPSS 21.0 software to perform descriptive statistics and correlation analysis of the variables. In this study, the reliability and validity of the variable measures needed to be examined in order to test the research questions posed. Skewness and kurtosis were also looked at to measure whether the data obtained had a normal distribution. Specifically, the mediating and moderating effects can be analyzed by means of specific models provided by PROCESS to assess the linkage of the conceptual framework and to analyze the influence relationships between the variables.

## 4. Results

### 4.1. Descriptive statistics

Of the 419 survey samples used,206 were males, and 213 were females. Also, we divided the age of users into six interval groups for testing. Moreover, the data shows that 43% of the sample is 18-29 years old, and 61.8% of the users are college students. The descriptive statistics of this study are summarized in Table 1.

**Table 1:** Respondent profiles (N =  419).

Measure		Frequency	Percent (%)
Gender	Male	206	49.2
	Female	213	50.8
Age(years)	Below 18 years old	6	1.4
	18–29 years old	180	43
	30–39 years old	136	32.5
	40–49 years old	56	13.4
	50–59 years old	26	6.2
	Above 60 years old	15	3.6
Academics	High school or below	15	3.6
	technical school	86	20.5
	College student	259	61.8
	Graduate school or above	56	13.4
	Others	3	0.7
Income	Below 3000	93	22.2
	3000–5000	21	5.0
	5000–7000	102	24.3
	7000–10000	121	28.9
	Above10000	82	19.6

Validity of the variables, we first conducted an exploratory factor analysis on the overall variables measured using SPSS statistical software. The initial factors were extracted using principal component analysis and then rotated using the maximum variance method in orthogonal rotation to retain factors with eigenvalues greater than or equal to 1 and factor loading greater than 0.7. According to the previous literature, Cronbach’s alpha greater than 0.7 is good [9]. The results showed that Cronbach’s alpha values for each variable ranged from 0.835 ~  0.903, all exceeding the acceptable level of 0.70, indicating a high level of internal consistency for statements measuring the same concept. To test the validity of the distinction between the constructs, a correlation coefficient analysis was conducted using SPSS, with the probability of significance assessed at the 0.05 and 0.01 levels and marked with an asterisk. Negative values between values indicate the presence of negative factors in the antecedent variables that affect perceived value. The results of the descriptive statistics and correlation analysis are given in Table 2.

**Table 2:** Correlations between variables.

Variables	IPR	RP	ANP	IF	INR	Cronbach’s alpha
IPR	1					0.903
RP	.263^**^	1				0.894
ANP	−.160^**^	−.138^**^	1			0.886
IF	−.130^**^	−.062	.363^**^	1		0.835
INR	.176^**^	.233^*^	−.066	−.081^**^	1	0.869

** *p* <  0.01,

* *p* <  0.05,

IPR, Infringement of privacy; RP, Risk perception; ANP, Anthropomorphism; IF, Informational; INR, Innovation resistance; Cronbach’s alpha: The reliability of internal consistency was evaluated with Cronbach’s alpha coefficients. This value is calculated using the following equation：α =  k/ (k−1) *  (1−Σs²/St²).

### 4.2. Analysis result

Table 3 shows the relationship between Infringement of privacy, Risk perception, and Innovation resistance. Specifically, the Infringement of privacy has no significant impact on Innovation resistance (β =  0.076, SE =  0.051, P =  0.136). On the other hand, we tested the moderated mediation model using model 21 of PROCESS with 95% confidence intervals and 5000 bootstrap samples, which were judged to be significant if the 95% confidence interval did not include 0, which has been standardized for all data. Specifically, the mediating role of Risk perception between Infringement of privacy and Innovation resistance was examined.

**Table 3:** Direct and indirect impact analysis.

Relationship	β	SE	95% CI Lower Upper		P	Results
	Direct effect					
IPR→INR	0.076	0.051	0.0240	0.1755	0.1362	
	Indirect effect					
IPR → RP→INR	0.014	0.063	0.004	0.028	Supported	

N =  419, bootstrapping randomly sampled 5,000 times. IPR, Infringement of privacy; RP, Risk perception; ANP, Anthropomorphism; IF, Informational; INR, Innovation resistance

The results of the study are shown in Table 3, where Risk perception has a significant mediating effect between Infringement of privacy and Innovation resistance (β =  0.014, 95% CI =  [0.004, 0.028], excluding 0). Therefore, hypotheses 1 are supported.

**Fig 2:**
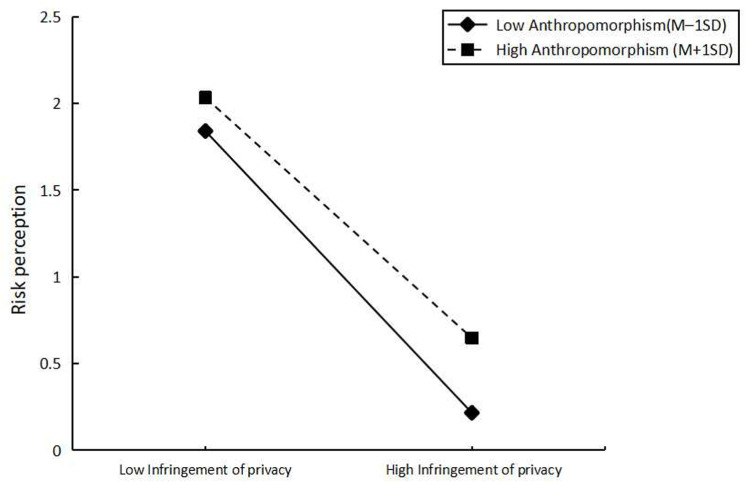
Showing moderation.

In addition, we tested the moderating effect of anthropomorphism by using model 21 in PROCESS. The results are shown in Table 4. Anthropomorphism produced a significant moderating effect between infringement of privacy and risk perception (β =  -0.119, t =  -3.677). To elucidate the above-mentioned moderating effects, we divided the anthropomorphism into two groups (M +  1 SD, M - 1 SD) (Fig 2). The results showed that infringement of privacy significantly affected users’ risk perception when anthropomorphism was low (mean - 1 SD) (b =  0.514, t =  6.044, p =  0.000). When anthropomorphism was high (mean +  1 SD), there was a significant effect of infringement of privacy and risk perception of AI voice assistants users (b =  0.125, t =  2.051, p =  0.041). Thus, the result found that anthropomorphism had a significant moderating effect on the infringement of privacy and Risk perception (β =  -0.119, 95% CI =  [-0.183, -0.056], excluding 0). This part of the analysis reveals that the interaction between perceived privacy invasion and anthropomorphism has a moderating effect on risk perception, have solved research question 1. Additionally, it was found that the degree of anthropomorphism has a negative influence on risk perception. In other words, despite concerns about privacy infringement, users may feel less risk when using AI voice assistants due to their perception of these assistants’ anthropomorphic characteristics.

**Table 4:** Moderation analysis.

	β	SE	95% CI Lower Upper		t	p	Results
Constant	1.029	0.604	−0.159	2.216	1.702	0.089	
IPR	0.812	0.158	0.501	1.123	5.130	0.000	
ANP	0.312	0.120	0.075	0.549	2.588	0.010	
R × ANP→RP	−0.119	0.032	−0.183	−0.056	−3.677	0.000	Supported

IPR, Infringement of privacy; RP, Risk perception; ANP, Anthropomorphism; IF, Informational; INR, Innovation resistance.

**Fig 3:**
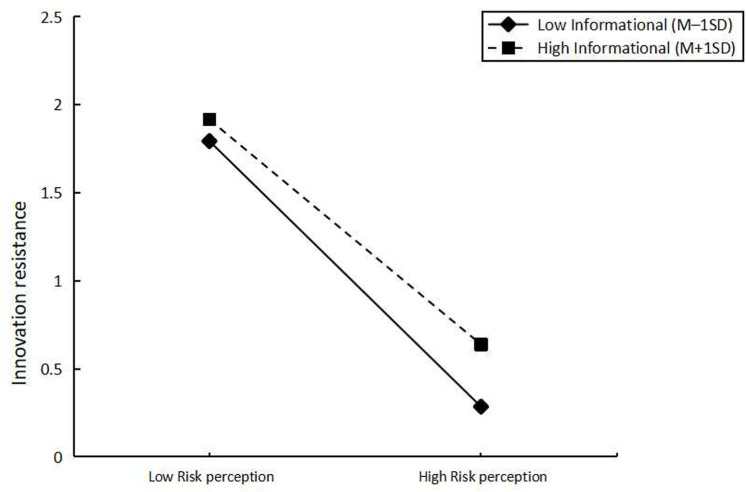
Showing moderation.

In addition, we tested the moderating effect of informational by using model 21 in PROCESS. The results are shown in Table 5. informational produced a significant moderating effect between risk perception and innovation resistance (β =  -0.116, t =  -3.573). To elucidate the above-mentioned moderating effects, we divided the informational into two groups (M +  1 SD, M - 1 SD) (Fig 3). The results showed that when informational was low (mean - 1SD), there was a significant effect on risk perception and innovation resistance of AI voice assistant users (b =  0.4076, t =  5.351, p =  0.000). When informational was high (mean +  1SD), there was a significant effect of risk perception and innovation resistance of AI voice assistant users (b =  0.0609, t =  1.009, p =  0.314). We found that informational had a significant moderating effect on risk perception and innovation resistance (β =  -0.116, 95% CI =  [-0.179, -0.052], excluding 0). The analysis in this part reveals that the interaction between risk perception and information moderates the effect on innovation resistance, addressing research question 2. Furthermore, we discovered that the degree of information negatively affects innovation resistance. This means that, despite risk perception, if the artificial intelligence voice assistant can offer valuable information to users, their level of innovation resistance will decrease.

**Table 5:** Moderation analysis.

	β	SE	95% CI Lower Upper		t	p	Results
Constant	1.039	0.629	−0.197	2.275	1.653	0.099	
IPR	0.076	0.051	−0.024	0.176	1.493	0.136	
RP	0.754	0.164	0.433	1.076	4.608	0.000	
IF	0.238	0.125	−0.008	0.484	1.903	0.058	
RP×IF→INR	−0.116	0.032	−0.180	−0.052	−−3.573	0.000	Supported

IPR, Infringement of privacy; RP, Risk perception; ANP, Anthropomorphism; IF, Informational; INR, Innovation resistance.

## 5. Discussion

This study investigates the relationship between privacy infringement, risk perception, and innovation resistance in the context of artificial intelligence voice assistants. The primary objective is to identify the key factors that influence users’ acceptance or resistance to this technological innovation, with a particular emphasis on the roles of anthropomorphic characteristics and the perception of information transfer. Through systematic research and rigorous analysis involving 419 participants, we obtained a series of significant results and insights. In constructing and validating the research model, we focused on evaluating the moderating effects of anthropomorphism and informatization on the relationship between privacy infringement and innovation resistance, as well as the mediating role of risk perception. The research clearly demonstrates that privacy infringement has a significant positive effect on risk perception. When users become acutely aware of the potential risks associated with using AI voice assistants, such as the threat of personal information leakage or location data exposure, their level of risk perception increases markedly. This finding aligns with previous studies in the domains of privacy and security. Research by Kim Jae Hui (2010) and Hwansoo Li et al. (2013) has shown a strong correlation between data collection practices and privacy risks [26,27]. This study further reinforces this relationship within the context of voice assistant usage. The findings underscore the importance of user sensitivity to privacy and security, as well as the pivotal role of risk perception in the decision-making process. Notably, privacy infringement does not exert a direct significant impact on innovation resistance; However, risk perception produced mediating effects.

This conclusion holds significant implications within the theoretical framework of innovation diffusion and user acceptance. It strongly demonstrates that consumers’ attitudes toward innovation are not solely influenced by the changes brought about by innovation itself, but are profoundly shaped by their risk perceptions during use. This finding aligns with the innovation acceptance theory proposed by Rogers (2003), which posits that individual acceptance of innovation is contingent upon a balance of various factors [11]. This study identifies risk perception as a core variable, offering valuable insights for technology developers and policymakers. Specifically, effectively managing user risk perception emerges as a crucial strategy for promoting the adoption of AI voice assistants, thereby achieving notable advancements in both theoretical understanding and practical application.

Further exploration of the moderating effects of personification and informationality revealed that personification significantly moderates the relationship between privacy infringement and risk perception. In the low-level range of anthropomorphism, the positive impact of privacy infringement on risk perception is extremely pronounced; however, as the level of anthropomorphism increases, this impact significantly diminishes. This finding aligns with the positive influence of anthropomorphism on user satisfaction and continued use intention identified in related research by Choi JS et al (2023) [30]. This study expands and deepens this relationship from the perspective of privacy risk perception, demonstrating that empowering voice assistants with anthropomorphic characteristics can effectively alleviate users’ privacy concerns and reduce risk perception. This offers new insights and a strong foundation for optimizing product design, further enriching and enhancing the user psychology research framework for artificial intelligence products. Additionally, informationality plays a significant moderating role between risk perception and innovation resistance. When the information supplied by the voice assistant is sufficient and accurately meets user needs, even if users possess a certain level of risk perception, their innovation resistance is significantly reduced. This result is supported by research in the fields of information systems and consumer behavior. For instance, studies by Zhang YN et al (2018) and Seo HG et al (2017) highlight the critical value of information in shaping consumer attitudes and behaviors [33,34]. This study accurately anchors its core role in addressing resistance to voice assistant innovation, strongly demonstrating that high-quality information services can effectively enhance users’ tolerance and acceptance of innovation, providing essential targets and practical guidelines for the optimization and upgrading of AI voice assistant functionalities. Compared with previous similar studies, this research presents significant advantages. The research design focuses on a core group of individuals aged 18-39, who are both highly receptive to and frequent users of AI products. This careful selection aims to accurately capture the psychological and behavioral dynamics of this demographic in relation to new technologies, ensuring that the findings are highly focused and closely aligned with actual application scenarios. This approach mitigates the uncertainty and lack of relevance often associated with broader sample sizes. Furthermore, the study provides direct and effective decision support for the precise and personalized development of AI voice assistants. In terms of variable consideration, it comprehensively integrates multidimensional key variables, including privacy infringement, risk perception, anthropomorphism, and information. By deeply analyzing the complex internal associations among these variables, the research constructs a systematic and comprehensive framework that transcends the limitations of previous studies, which often focused on single variables or simplistic associations. This holistic approach reveals the underlying logic of user acceptance of AI voice assistants in a thorough manner. Ultimately, it offers systematic and comprehensive theoretical and methodological examples for future research, effectively advancing the field from a fragmented state to a more systematic development stage.

## 6. Limitations and future research

There are certain limitations to the investigation. First and foremost, the research sample and the extent of generalizability of the research findings are restricted due to the exclusive focus on survey data from China. To enhance the comprehensiveness and inclusiveness of future studies, it would be beneficial to compare the disparities in users’ perceptions of the value of AI voice assistants across various countries. At the same time, this study focuses on 18 to 39 age group, a demographic that typically demonstrates a higher acceptance of technology and frequent utilization of AI products, thereby offering more targeted insights. Although the sample size is limited, the study’s design, data collection, and analysis adhered to rigorous academic standards, yielding valuable findings with significant academic and practical implications. In the future, as the range of applications and users of AI products broadens, the generalizability of the results could be improved by expanding the sample to include diverse age groups, genders, and cultural backgrounds. Secondly, as technology continues to advance and systems are regularly updated, there is ample room for further exploration in areas such as AI voice assistant user experience. Particularly, delving into the impact of the combination of anthropomorphism and individuals’ personal traits on their sustained usage of artificial intelligence voice assistants is a noteworthy topic for deliberation. Moreover, this study solely conducted a generic assessment of information without an intricate categorization of its content. In subsequent research, it would be fruitful to examine the moderating effects of distinct information types on risk perception and resistance to innovation. Additionally, comprehending the concerns of potential users who have yet to adopt or decline the use of AI voice assistants is imperative for the future advancement of the industry. This understanding will facilitate the expansion of the target market and enable sustainable growth in current market conditions.

## 7. Conclusions

Research indicates that risk perceptions mediate the relationship between privacy violations and resistance to innovation. This finding is significant as it underscores the indirect pathway through which privacy concerns shape resistance to new technologies. Additionally, this paper identifies anthropomorphism and informativeness as key moderating variables. Specifically, it demonstrates that these factors can alleviate the perceived risks associated with AI voice assistants, thereby diminishing resistance to innovation. By emphasizing user psychology, the research offers valuable insights into the design and development of AI voice assistants, highlighting the necessity of addressing user concerns regarding privacy and risk.

With the continuous advancement of artificial intelligence and machine learning, machines can now offer personalized advice and engage in communication through social robots. Nonetheless, the growing reliance on AI also raises concerns regarding online privacy infringement. To tackle these concerns, enterprises must conduct comprehensive tests to identify and address potential issues or risks associated with their products or services. By prioritizing the enhancement of product quality and design, developers can effectively decrease consumers’ resistance. Furthermore, this study delves into the notion of anthropomorphism, which involves attributing human-like characteristics and perspectives to non-human entities; the moderation role of anthropomorphism in the domain of robots and interactive services is also noteworthy. Additionally, the intelligent recommendation feature of big data and intelligent algorithms allows AI voice assistants to offer consumers personalized insights and an abundance of cross-domain knowledge. Such information enhances the perceived value of these assistants and diminishes resistance toward innovation. To summarize, ensuring secure and seamless interactions between users and AI voice assistants while enabling the ethical collection of valuable information for developers and businesses is imperative for their future development.
